# New insights into the plastome evolution of Lauraceae using herbariomics

**DOI:** 10.1186/s12870-023-04396-4

**Published:** 2023-08-10

**Authors:** Zhi Yang, David Kay Ferguson, Yong Yang

**Affiliations:** 1 Co-Innovation Center for Sustainable Forestry in Southern China, College of Biology and the Environment, Nanjing Forestry University, 159 Longpan Rd, Nanjing, 210037 China; 2 Department of Paleontology, University of Vienna, Vienna, Austria

**Keywords:** Evolution, Herbariomics, Lauraceae, Phylogenomics, Plastome

## Abstract

**Background:**

The family Lauraceae possesses ca. 50 genera and 2,500–3,000 species that are distributed in the pantropics. Only half of the genera of the family were represented in previously published plastome phylogenies because of the difficulty of obtaining research materials. Plastomes of Hypodaphnideae and the *Mezilaurus* group, two lineages with unusual phylogenetic positions, have not been previously reported and thus limit our full understanding on the plastome evolution of the family. Herbariomics, promoted by next generation sequencing technology, can make full use of herbarium specimens, and provides opportunities to fill the sampling gap.

**Results:**

In this study, we sequenced five new plastomes (including four genera which are reported for the first time, viz. *Chlorocardium*, *Hypodaphnis*, *Licaria* and *Sextonia*) from herbarium specimens using genome skimming to conduct a comprehensive analysis of plastome evolution of Lauraceae as a means of sampling representatives of all major clades of the family. We identified and recognized six types of plastomes and revealed that at least two independent loss events at the IR-LSC boundary and an independent expansion of SSC occurred in the plastome evolution of the family. *Hypodaphnis* possesses the ancestral type of Lauraceae with *trnI-CAU*, *rpl23* and *rpl2* duplicated in the IR regions (Type-I). The *Mezilaurus* group shares the same plastome structure with the core Lauraceae group in the loss of *trnI-CAU*, *rpl23* and *rpl2* in the IRa region (Type-III). Two new types were identified in the *Ocotea* group: (1) the insertion of *trnI-CAU* between *trnL-UAG* and *ccsA* in the SSC region of *Licaria capitata* and *Ocotea bracteosa* (Type-IV), and (2) *trnI-CAU* and pseudogenizated *rpl23* inserted in the same region of *Nectandra angustifolia* (Type-V). Our phylogeny suggests that Lauraceae are divided into nine major clades largely in accordance with the plastome types. The Hypodaphnideae are the earliest diverged lineage supported by both robust phylogeny and the ancestral plastome type. The monophyletic *Mezilaurus* group is sister to the core Lauraceae.

**Conclusions:**

By using herbariomics, we built a more complete picture of plastome evolution and phylogeny of the family, thus providing a convincing case for further use of herbariomics in phylogenetic studies of the Lauraceae.

**Supplementary Information:**

The online version contains supplementary material available at 10.1186/s12870-023-04396-4.

## Background

Lauraceae, belonging to the Laurales of magnoliids, contain ca. 50 genera and 2,500–3,000 species [1–3]. Species of this family are mostly woody with exception of the herbaceous parasite *Cassytha* and widely distributed in tropical and subtropical regions [4]. Tall tree species are dominant in the evergreen broad-leaved forests of the tropics and important in maintaining the local communities [4–7]. In addition, many Lauraceae species are valuable economically, as a source of medicines, excellent timber, fruits, spices, and perfumes [4, 8, 9].

The phylogeny of the family Lauraceae remains poorly resolved because of the low resolution of molecular markers and inadequate sampling of species. Over the past two decades, published phylogenetic studies of Lauraceae were mainly based on single or multiple molecular markers [2, 10–18]. Due to low divergence of commonly used markers, inter- and intrageneric phylogenetic relationships within the family have not been fully resolved [19–21].

**Fig. 1:**
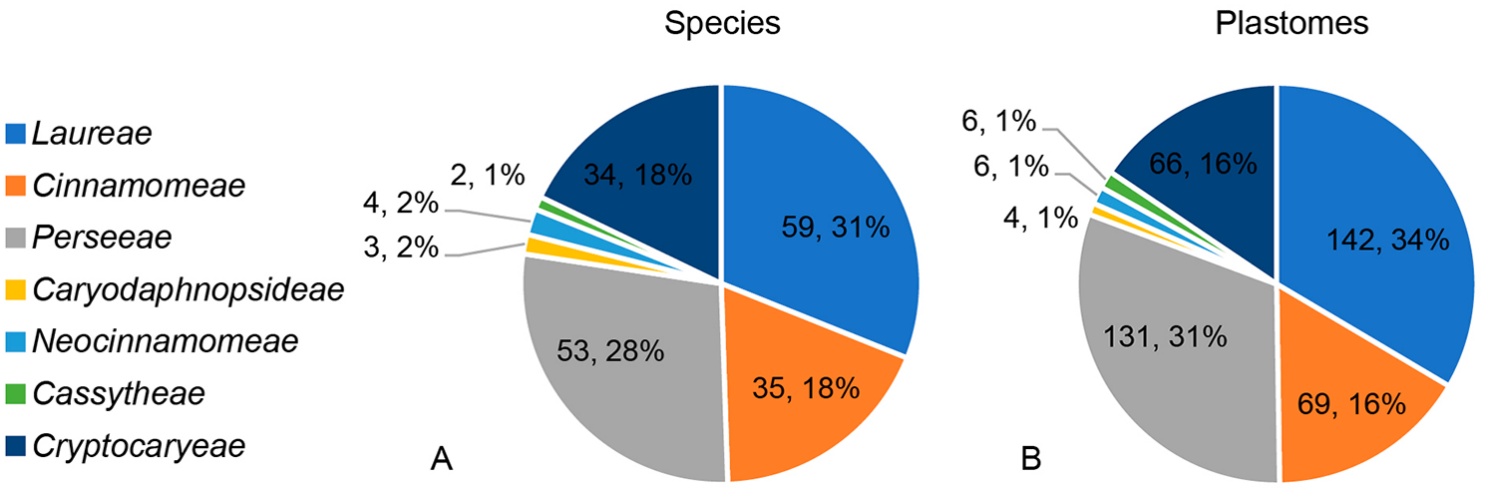
Visualization of available plastomes of Lauraceae in NCBI. **(A)** The systematic distribution of available species, the species number and relative percentage of each clade are shown in the pie charts; **(B)** The systematic distribution of available plastomes, the plastome number and relative percentage of each clade are shown in the pie charts. Different clades are indicated by different colors

Plastome sequences have been successfully used for inferring phylogeny of green plants at different taxonomic levels owing to rich sequence variation [22–25]. Plastome sequences have also been used to resolve inter- and intrageneric phylogeny of the family Lauraceae [19, 20, 24, 26–28]. At the family level, both Song et al. [19] and Liu et al. [20] recognized nine clades of Lauraceae (i.e., Hypodaphnideae, Cryptocaryeae, Caryodaphnopsideae, Neocinnamomeae, Cassytheae, *Mezilaurus* group, Perseeae, Cinnamomeae and Laureae), though they did not sample two of them in their phylogenomic studies, i.e., *Hypodaphnis* and the *Mezilaurus* group. Insufficient sampling of important lineages has been an obstacle to a better understanding of the plastome evolution of the family Lauraceae. Plastomes of 190 species of 27 genera of Lauraceae are available in NCBI (Table S1; accessed 22 March 2022), over 90% of them belong to Cryptocaryeae and the core Lauraceae group, and most of them are from Asia (Fig. 1) [29]. Neotropical species of Cinnamomeae remain poorly represented, only one plastome of *Nectandra* and seven plastomes of *Ocotea* were sequenced [26, 29]. In particular, the African *Hypodaphnis* and the American *Mezilaurus* group represent evolutionary distinct lineages of Lauraceae but are still lacking in plastome studies: the genus *Hypodaphnis* is the earliest diverged lineage in the family Lauraceae (Hypodaphnideae), and the *Mezilaurus* group is sister to the core Lauraceae [2, 10, 11]. This sampling bias is largely attributable to the unavailability of research materials.

Content, structure, and gene organization of plastomes are important in understanding evolutionary relationships of plants [30, 31]. Plastomes of Lauraceae show a relatively conserved quadripartite structure, and consist of 128–130 genes except *Cassytha* with only 113 genes [26, 29, 32]. Recent studies have suggested that at least four different types of plastomes were existing in the family Lauraceae according to variation of *ycf2*-*rpl2* regions at the IR-LSC boundary [26, 28, 29, 32]. The plastome of Cryptocaryeae lost the *rpl2* gene in the IRb. Plastomes of Caryodaphnopsideae, Neocinnamomeae and the core Lauraceae group lost a segment of *ycf2* and total *trnI-rpl23-rpl2* region in the IRa. The parasitic genus *Cassytha* is unique in losing the entire IR region. The fourth type, only found in plastomes of *Caryodaphnopsis henryi* and a sample of *Cinnamomum chartophyllum* (synonym of *Camphora chartophylla*), contained two copies of *rpl2* in the IR regions [26, 28]. At least two independent events caused by IR reduction might have occurred in the plastome evolution of Lauraceae [26, 32].

Herbaria are a “treasure trove”, harboring thousands of specimens with accurately identified materials and enormously relevant information [33, 34]. Museum specimens can improve material availability and overcome sampling biases in phylogenetic studies if they can be used in sequencing studies. However, it is difficult to obtain sequences using Sanger sequencing method because museum DNA is highly degraded and fragmented, and DNA extraction and gene amplification of Lauraceae are also challenging because of rich polysaccharides and polyphenols in plant tissues [1, 35]. Driven by next-generation sequencing (NGS) technology, herbariomics (Herbarium genomics) is a promising field [35, 36]. By using herbarium specimens, this new approach can largely solve the problem of sampling bias and taxonomic identification on the one hand, and is a cost-efficient and time-saving approach on the other hand [36, 37]. Herbarium specimens have rarely been used in phylogenomic studies of the family Lauraceae though plastomes were successfully obtained from specimens of *Phoebe neurantha* and *Cin. bodinieri* preserved for 79 years and 59 years, respectively [38].

In this study, we successfully obtained five plastomes representing five genera (*Licaria*, *Ocotea, Chlorocardium*, *Sextonia* and *Hypodaphnis*) from herbarium specimens, and filled the sampling gap of *Hypodaphnis* and the *Mezilaurus* group. We tested the applicability of herbariomics in phylogenomic studies of Lauraceae and explored the plastome evolution of the family.

## Results

### Characteristics of the five newly sequenced plastomes

**Fig. 2:**
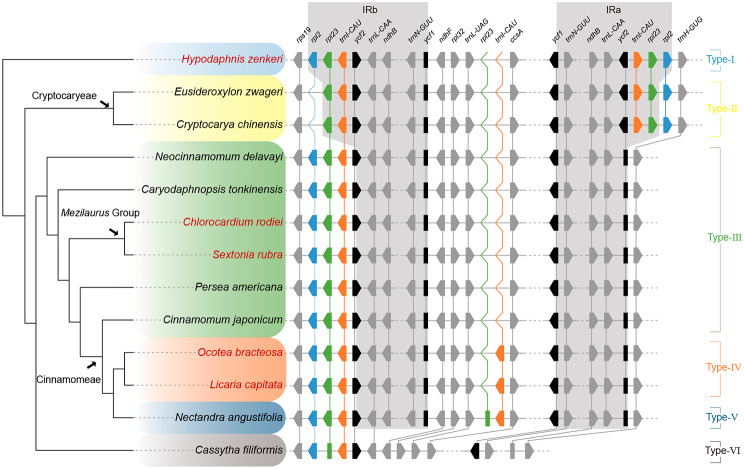
Structural variation and evolution of plastomes of Lauraceae. Five newly sequenced plastomes are colored in red. Gene loss/gain and IR boundary are shown in the right panel. Transcriptional orientations of genes are indicated excepting pseudogenes. Three unstable genes (*rpl2*, *rpl23* and *trnI-CAU*) are shown in different colors, while *ycf1* and *ycf2* which occur near IR boundary are colored in black. Orthologous genes are linked with vertical lines. Genes and their relative positions are not drawn to scale

Five newly sequenced plastomes from herbarium specimens were successfully assembled to complete the circle. All plastomes shared the typical quadripartite structure with two copies of inverted repeat (IRa, IRb) regions, which separated the large single copy region (LSC) and small single copy region (SSC), respectively (Fig. S1). *Licaria capitata*, *Ocotea bracteosa*, *Chlorocardium rodiei* and *Sextonia rubra* show little variation in length and GC content of complete plastome sequences and LSC, SSC and IR regions (Table 1). *Hypodaphnis zenkeri* was distinct from the other four plastomes with a longer sequence (157,231 bp vs. 151,752 bp–153,108 bp), longer IR region (25,518 bp vs. 19,884 bp–20,102 bp), longer SSC region (19,399 bp vs. 17,942 bp–19,065 bp), and shorter LSC region (86,796 bp vs. 93,585 bp–93,899 bp). Meanwhile, lower GC content was detected in the complete plastome (39.0% vs. 39.2–39.3%), LSC (37.8% vs. 38–38.1%) and IR (43.2% vs. 44.4–44.5%) of *H. zenkeri* than in the other four species. These plastomes contained about 128–131 genes, including 84–86 protein-coding genes, 36–37 tRNA genes, and eight rRNA genes (Table 1). *Chlorocardium rodiei* and *S. rubra* possessed three coding genes (*ndhB*, *rps7* and *rps12*), two truncated genes (*ycf1* and *ycf2*), four rRNA genes (*rrn4.5*, *rrn5*, *rrn16* and *rrn23*) and six tRNA genes (*trnA-UGC*, *trnI-GAU*, *trnL-CAA*, *trnN-GUU*, *trnR-ACG* and *trnV-GAC*) duplicated in the IR regions (Fig. 2, S2). *Hypodaphnis zenkeri* contained three more duplicated genes (*rpl23*, *rpl2*, and *trnI-CAU*) and a complete *ycf2* gene in the IRa unlike the other species (Fig. 2, S1). Notably, *L. capitata* and *O. bracteosa* possessed one more *trnI-CAU* gene in the SSC region appearing in the other species (Fig. 2, S1, Table 1). This unique variation was confirmed by a PCR test using gene-specific primers (Fig. S2B). Totally 18 genes in the five plastomes were found to possess introns, included 12 protein coding genes (*clpP1*, *ycf3*, *atpF*, *ndhA*, *ndhB*, *petB*, *petD*, *rpl2*, *rpl16*, *rpoC1*, *rps12*, and *rps16*), and six tRNA genes (*trnA-UGC*, *trnG-UCC*, *trnI-GAU*, *trnK-UUU*, *trnL-UAA*, and *trnV-UAC*). Among these 18 genes, only *clpP1* and *ycf3* contained two introns while the other 16 genes possessed only one intron. The gene *rps12* was trans-spliced with the 5’end and the duplicated 3’end located in the LSC and IR regions, respectively.

**Table 1:** Characters of five newly sequenced plastomes

Characteristics	*Licaria capitata*	*Ocotea bracteosa*	*Chlorocardium rodiei*	*Sextonia rubra*	*Hypodaphnis zenkeri*
Total cpDNA size (bp)	152,649	152,748	152,664	151,752	157,231
Length of large single copy (LSC) region (bp)	93,713	93,725	93,899	93,585	86,796
Length of small single copy (SSC) region (bp)	18,978	19,065	18,997	17,942	19,399
Length of inverted repeat (IRs) region (bp)	19,979	19,979	19,884	20,102	25,518
Total GC content (%)	39.2%	39.2%	39.3%	39.3%	39%
LSC-GC content (%)	38%	38%	38.1%	38%	37.8%
SSC-GC content (%)	33.8%	34.1%	34%	34.4%	33.5%
IR-GC content (%)	44.4%	44.5%	44.5%	44.5%	43.2%
Total number of genes (unique)*	129 (113)	129 (113)	128 (113)	128 (113)	131 (113)
Total number of proteins encoding genes (unique)	84 (79)	84 (79)	84 (79)	84 (79)	86 (79)
Total number of tRNA genes (unique)	37 (30)	37 (30)	36 (30)	36 (30)	37 (30)
Total number of rRNA genes (unique)	8 (4)	8 (4)	8 (4)	8 (4)	8 (4)

Note: * including pseudogene

**Fig. 3:**
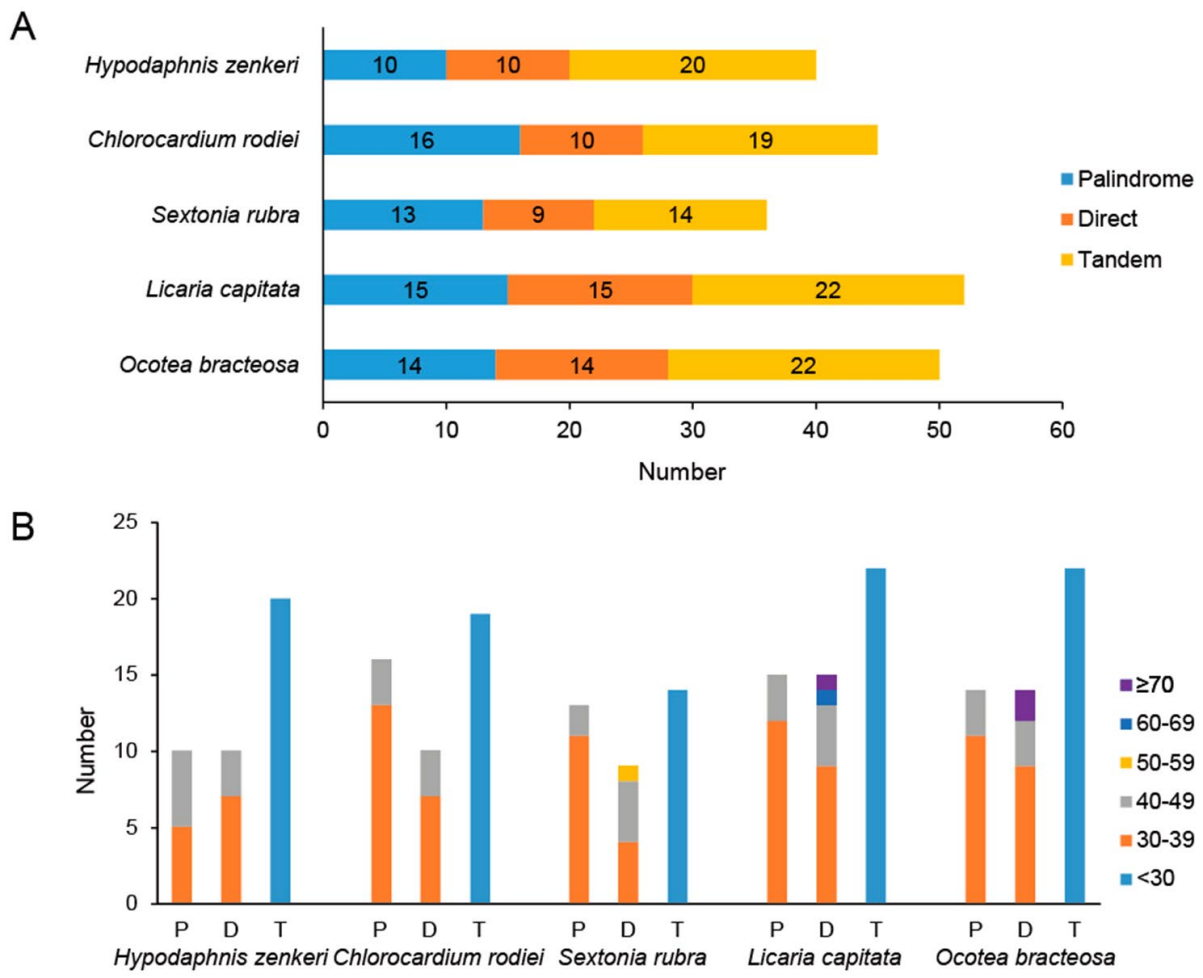
Repeats of the five newly sequenced plastomes. **A**. Number of three types of repeats; **B**. Length of three types of repeats. P = Palindrome repeat, D = Direct repeat, T = Tandem repeat

On average, 45 repeats were found in all newly sequenced plastomes (Fig. 3, Table S2). *Licaria capitata* contained the highest number of repeats (52). Three types of repeats including tandem, palindromic and direct repeats were identified. The tandem repeats had the highest proportion (ca. 43.5%), followed by the palindromic repeats (ca. 30%), and the direct repeats (ca. 26.5%). All tandem repeats were shorter than 30 bp, while all palindromic and direct repeats were longer than 30 bp (Fig. 3, Table S3). The longest repeat was 153 bp and belonged to a direct repeat, which was found in *L. capitata* and *O. bracteosa*, and associated with the *trnI-CAU* gene.

**Fig. 4:**
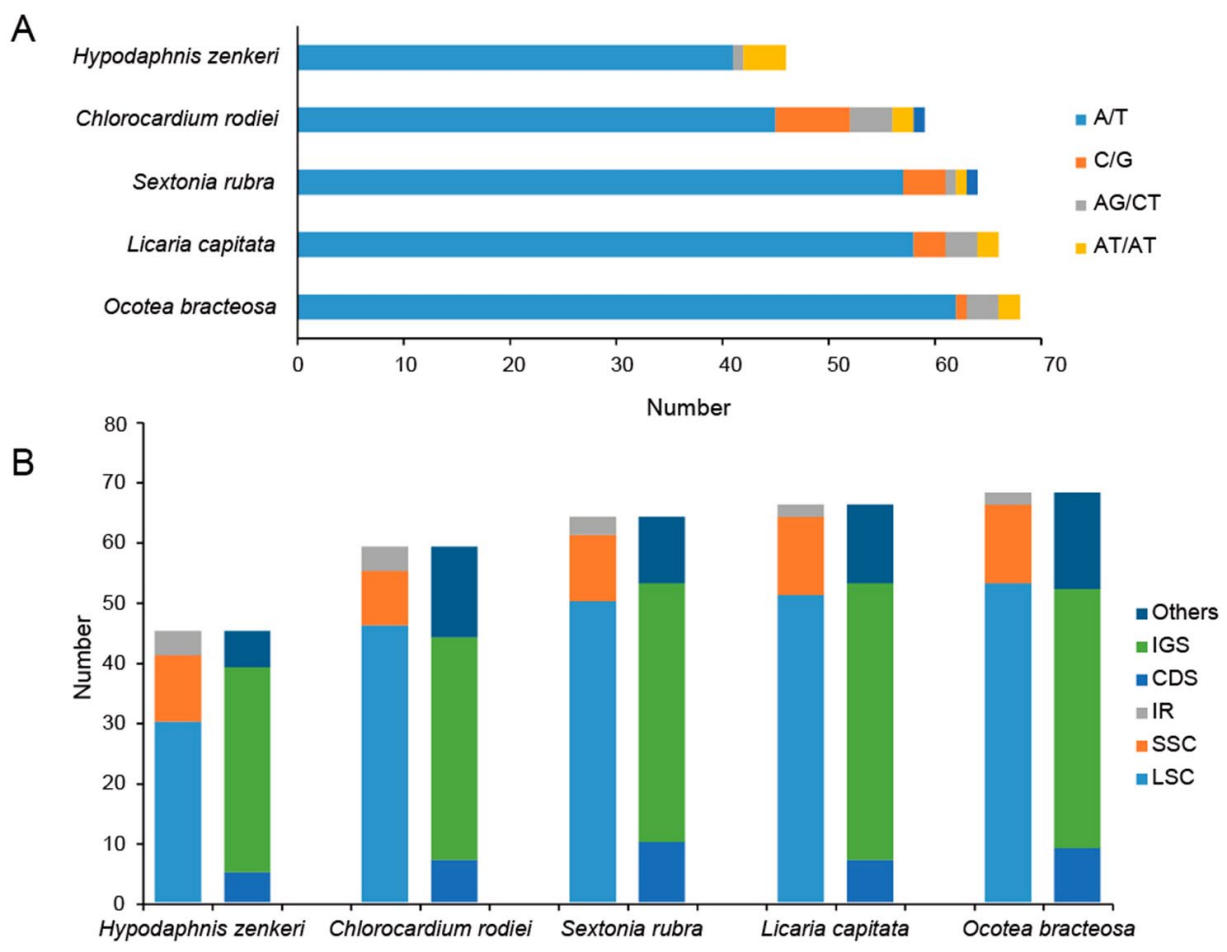
SSR analysis of the five newly sequenced plastomes. **A**. Simple sequence repeat unit composition; **B**. The distribution of repeats in the large single copy (LSC) region, the small single copy (SSC) region, the inverted repeat regions (IRs), the intergenic spacer regions (IGS), the coding DNA sequences (CDS) and the others

SSRs were detected in all species, classified into three types, i.e. mono-, di- and trinucleotides repeats (Fig. 4A, Table S4). *Hypodaphnis zenkeri* contained the least number of SSRs. Trinucleotide repeats were only found in *C. rodiei* and *S. rubra*. Mononucleotide repeats were the most common SSRs (up to 91.7% of total) in which A/T monomers occupied 94.6%, while other types of SSRs were rare. Meanwhile, most SSR loci were scattered in the LSC (30–53), rarely found in SSC (9–13) and IR (2–4) regions. IGS (34–46) contained more SSRs than CDS and the others (Fig. 4B, Table S5).

### Plastome variation of Lauraceae

The plastomes of Lauraceae contained at least six major types according to the varied number and position of *rpl2*, *rpl23* and *trnL-CAU* genes (Fig. 2). Type-I was characteristic of the Hypodaphnideae with *rpl2*, *rpl23* and *trnI-CAU* located in both IR regions. Type-II was restricted to the Cryptocaryeae with one copy of *rpl2* missing due to contraction of the IRb boundary. In contrast, Type-III plastomes lost not only *rpl2* but also *rpl23*, *trnI-CAU* and part of *ycf2* due to contraction of the IRa boundary. This type was found in the remaining laurel species excepting the unique *Cassytha filiformis* whose IR was lost (Type-VI), and three American species from the *Ocotea* group. *Ocotea bracteosa* and *L. capitata* displayed a new type (Type-IV) with the plastomes gaining another copy of *trnI-CAU* near *ccsA* in SSC region compared with Type-III. Moreover, our re-annotation of the plastome of *Nectandra angustifolia* showed that it not only acquired an additional copy of *trnI-CAU* but also had a pseudogenizated *rpl23* gene inserted between *trnI-CAU* and *ccsA* in the SSC region, which was defined as Type-V.

**Fig. 5:**
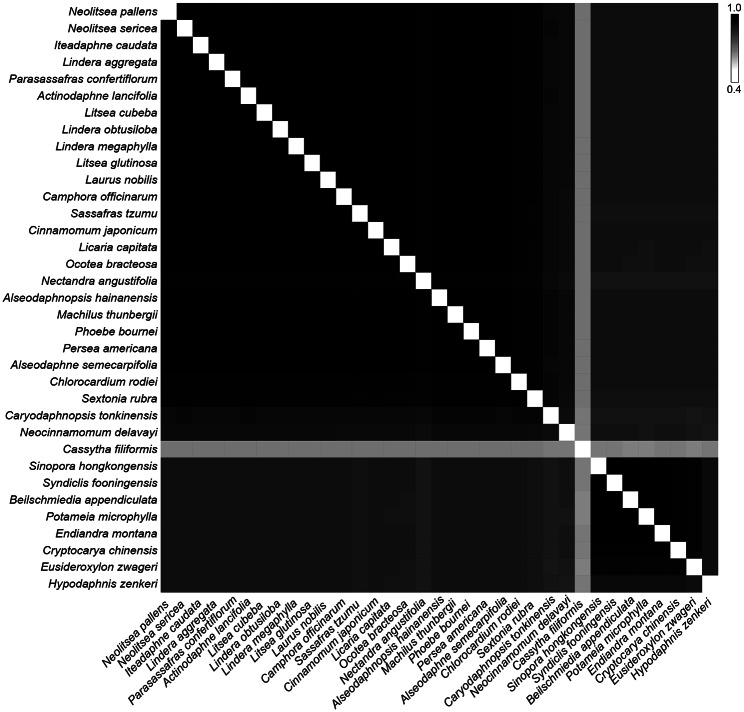
Similarity plot based on pairwise comparison of plastomes from the untrimmed whole-genome alignment. Similarity scores are color-coded from white (40% sequence identity) to black (100% sequence identity)

Pairwise alignments of sampled plastome sequences of Lauraceae showed a high similarity of over 84.7% (Fig. 5, Table S6), except for the parasitic *Cas. filiformis* displaying extremely low similarity (63.5–65.5%) to other genera. Two clusters were established based on similarity. One cluster comprised the core Lauraceae and the *Mezilaurus* group, which indicated higher similarity (≥ 94.0%) with one another; almost all species of the core Lauraceae displayed a pairwise similarity of over 98.0%. Notably, *N. angustifolia* had the lowest pairwise similarity range from 96.2 to 97.3% among the core Lauraceae. The other cluster consisted of the Cryptocaryeae with pairwise similarity over 94.0%. *Caryodaphnopsis tonkinensis*, *Neocinnamomum delavayi* and *H. zenkeri* were relatively independent and showed similarity lower than 92%, 90.1% and 89.6% with other species of Lauraceae, respectively.

To investigate the plastome variation at the gene level, we calculated the percentages of variable characters for coding and non-coding regions of all sampled species. In total, coding regions were more conservative than noncoding regions (Fig. S3C). There were 14 coding regions exhibiting high variation (Fig. S3A): *matK*, *rps16, rpoC2*, *accD*, *rpl20*, *rpoA*, *rps8*, *rpl22*, *rpl2, rpl23, ycf2*, *ycf1*, *rpl32* and *ccsA* (the percentage of variation > 20%). The SSC region contained only two genes (*ycf1* and *rpl32*) with the percentage of variation over 30%. Seven non-coding regions exhibited high variation (Fig. S3B): *psbA_trnH-GUG*, *trnG-UCC_trnR-UCU*, *rpl2_rps19*, *trnI-CAU_ycf2*, *rpl32_trnL-UAG*, *ccsA_trnL-UAG*, *rps15_ycf1* (the percentage of variation > 40%). Highly divergent regions were mainly distributed in IR boundaries. The region between *trnI-CAU* and *ycf2* showed the highest variation at 59%.

### Phylogenomics of Lauraceae

The complete plastomes and protein-coding genes of 35 species of Lauraceae were used to reconstruct phylogenetic trees. The two aligned data matrices were 150,930 bp and 66,843 bp long, respectively, and contained 13,907 bp (9.2%) and 5,731 bp (8.6%) parsimony informative sites, respectively.

**Fig. 6:**
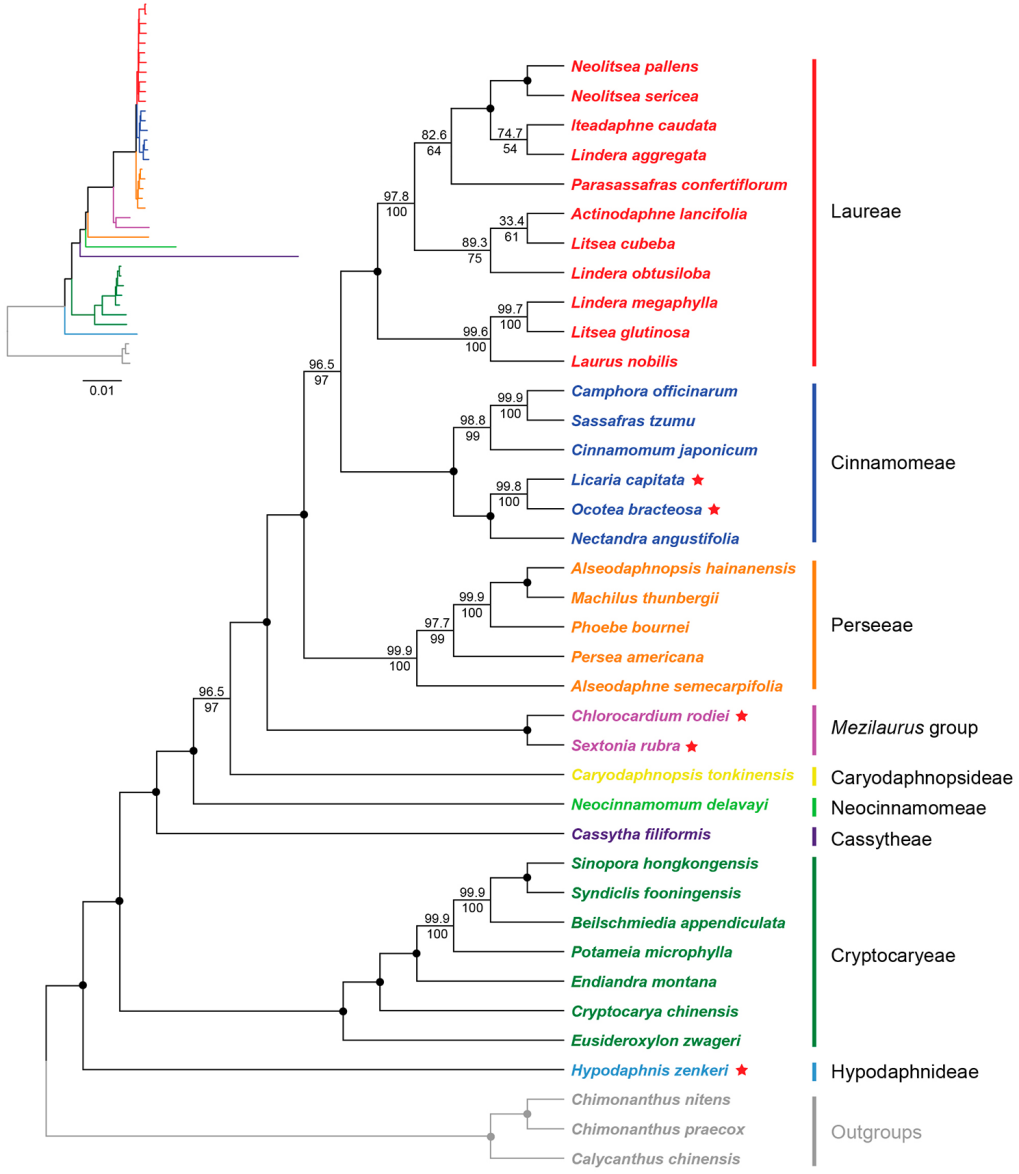
Maximum-likelihood (ML) tree inferred from CDS genes. Different tribal clades are highlighted with different colors. Five newly sequenced species are indicated with a red star. Each branch is assigned with UFBoot and SH-aLRT supports that are indicated above and below the line, respectively. The clades with 100% support for both tests are indicated by a black circle at the node. The phylogenetic tree with branch length is shown on the upper left

Both ML trees indicate that the Lauraceae are divided into nine clades (Fig. 6, S4) corresponding to the eight previously described tribes (Hypodaphnideae, Cryptocaryeae, Caryodaphnopsideae, Neocinnamomeae, Cassytheae, Perseeae, Cinnamomeae and Laureae) and the *Mezilaurus* group. *Hypodaphnis zenkeri* was confirmed to be the earliest diverged lineage with 100% support in both ML trees. Then Cryptocaryeae, Caryodaphnopsideae, Neocinnamomeae, Cassytheae, *Mezilaurus* group, Perseeae, Cinnamomeae and Laureae diverged in order with 100% support excepting Caryodaphnopsideae which received relatively lower support (CDS: UFBoot = 96.5% and SH-Alrt = 97%, CPG: UFBoot = 98.8% and SH-Alrt = 97%). The newly sampled *C. rodiei* and *S. rubra* formed a clade (CDS-CPG = 100%) representing the *Mezilaurus* group and sister to a clade consisting of Laureae, Cinnamomeae and Perseeae (the core Lauraceae). Cinnamomeae were separated into two subclades that are distributed in Asia and America respectively (CDS-CPG = 100%). The Asian clade (CDS: UFBoot = 98.8% and SH-Alrt = 99%, CPG = 100%) includes *Cinnamomum* and a clade consisting of *Sassafras* and *Camphora* (CDS: UFBoot = 99.9% and SH-Alrt = 100%, CPG = 100%). The American clade (CDS-CPG = 100%) included three genera, *Nectandra* was sister to a group consisting of *Licaria* and *Ocotea* (CDS: UFBoot = 99.8% and SH-Alrt = 100%, CPG = 100%). Two phylogenetic trees displayed similar topologies except for a minor difference in the support of Laureae. In the CPG phylogeny, *Lindera aggregata* was sister to a small clade encompassing *Neolitsea pallens*, *Neo. sericea* and *Iteadaphne caudata* (UFBoot-SH-Alrt = 100%); *Actinodaphne lancifolia* was the sister group of *Lin. obtusiloba* and *Litsea cubeba* (UFBoot = 98.9% and SH-Alrt = 92%). Unlike the CPG phylogeny, *Lin. aggregata* formed a clade with *I. caudata* (UFBoot = 74.7% and SH-Alrt = 54%), which was sister to *Neo. pallens* and *Neo. sericea*; *Lin. obtusiloba* was the sister group of *A. lancifolia* and *Lit. cubeba* (UFBoot = 89.3% and SH-Alrt = 75%) in the CDS phylogeny. Both *Lindera* and *Litsea* were polyphyletic in our study.

### Divergence time of Lauraceae

**Fig. 7:**
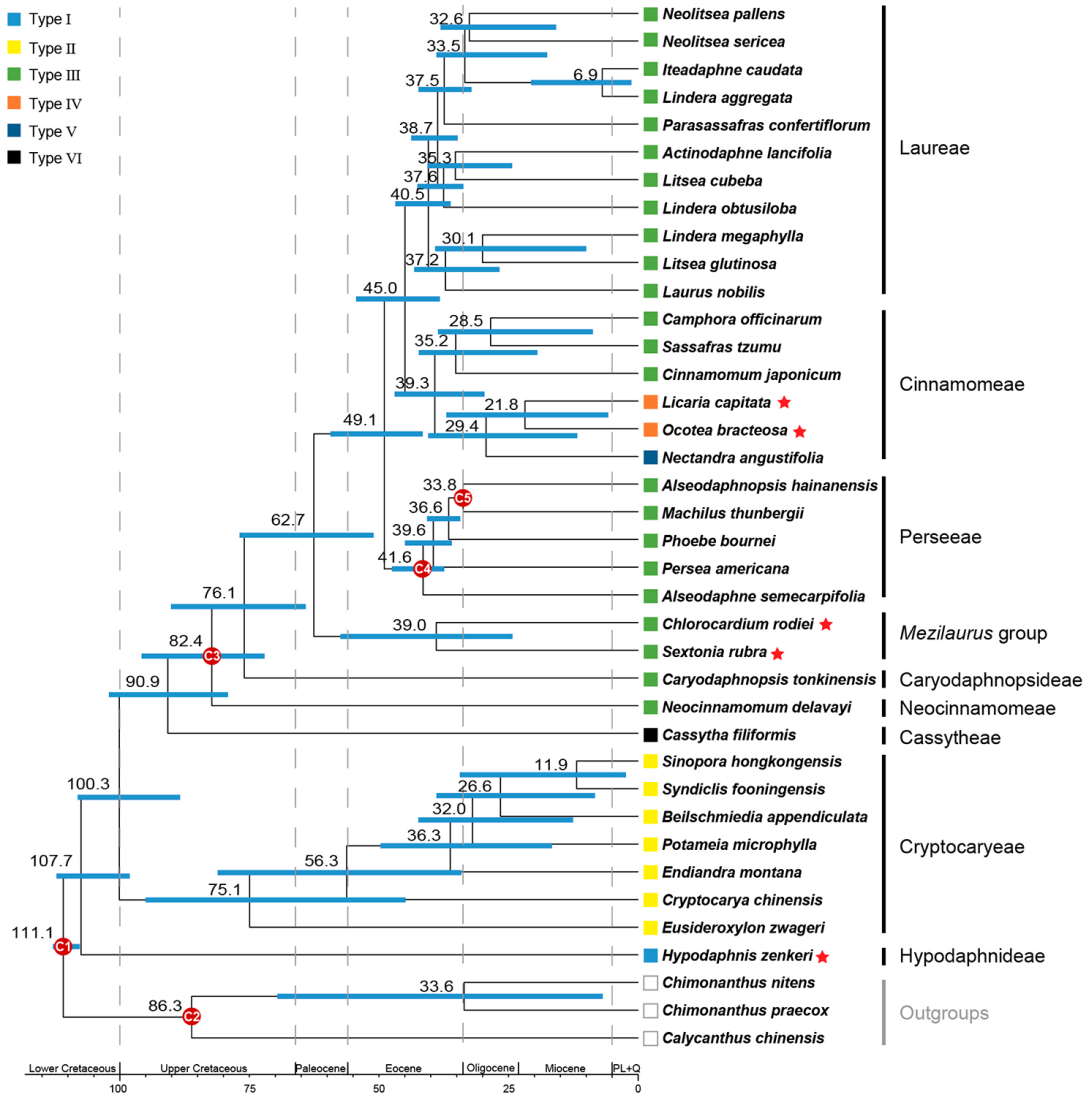
The chronogram of Lauraceae using MCMCtree. Blue bars on the nodes indicate the 95% HPD, mean age of each node is indicated above the bar, calibrating nodes are shown by red circles. Five newly sequenced species are indicated with a red star. For geologic timescale and subdivisions, PL + Q is abbreviated for Pliocene and the Quaternary. Six types of chloroplast genomes are indicated by rectangles with different colors on tip nodes

The divergence time between Lauraceae and Calycanthaceae was estimated to be 111.1 mya (95% highest posterior density (HPD): 107.9–113.1 mya) in the Albian during the Early Cretaceous, and the estimated crown age for Lauraceae was ca. 107.7 mya (95% HPD: 98.3–112.4 mya) (Fig. 7). The Cryptocaryeae diverged from the remaining laurels around 100.3 mya (95% HPD: 88.5–108.3 mya), and the crown age of Cryptocaryeae was ca. 75.1 mya (95% HPD: 44.9–95.2 mya) around the K-T boundary. Cassytheae diverged from its sister clade around 90.9 mya (95% HPD: 79.3–102.3 mya), followed by the Neocinnamomeae and Caryodaphnopsideae with estimated divergence ages of 82.4 mya (95% HPD: 72.2–96 mya) and 76.1 mya (95% HPD: 64.2–90.3 mya) in the Late Cretaceous, respectively. The divergence between the *Mezilaurus* group and the core Lauraceae occurred in the early Paleocene, ca. 62.7 mya (95% HPD: 51.1–77mya). The crown age of the *Mezilaurus* group was inferred to be 39 mya (95% HPD: 24.3–57.5 mya) during the Late Eocene. The earliest divergence between Perseeae and the remaining clade of the core Lauraceae occurred around 49 mya (95% HPD: 41.6–59.5 mya) in the Early Eocene, followed by the split of Cinnamomeae and Laureae, estimated to be around 45 mya (95% HPD: 38.3–54.5 mya). The estimated crown age for Perseeae, Cinnamomeae and Laureae was 41.6 mya (95% HPD: 37.4–47.5 mya), 39.3 mya (95% HPD: 29.7–47 mya) and 40.5 mya (95% HPD: 36.2–46.9 mya), respectively.

## Discussion

### Structural variation of plastomes in Lauraceae

By supplementing the five newly sequenced plastomes, we had representatives of all the nine clades of Lauraceae to achieve a more comprehensive knowledge of the plastome structure of the family. Plastomes of the family Lauraceae are conserved with a high sequence similarity no less than 84.7% between clades (excepting *Cassytha*; Fig. 5; Table S6), but gain and loss of DNA fragments do provide characters to classify the plastomes of the family into six types which are largely congruent with intra-familial phylogenetic relationships (Fig. 2). Four of these six types have been reported in recent studies [26, 32], corresponding to the Types I, II, III and VI recognized in this study (Fig. 2); here we recognize two new plastome types in the *Ocotea* group, i.e., Type-IV and Type-V (Fig. 2).

*Hypodaphnis* is the most primitive branch in the Lauraceae, and its plastome had not been reported. This genus has the Type-I plastome (Fig. 2), which contains two copies of *rpl2* in the IR regions, and possesses the largest number of genes (131) and protein coding genes (86) in the family Lauraceae (Table 1) [29, 32]. In addition, the plastome of *Hypodaphnis* has fewer SSRs and the lowest GC content in the family Lauraceae (Table 1, S4) [29]. This type of plastome was reported as an exceptional variation in Song et al. [26] and Xiao and Ge [28].

The *Mezilaurus* group had not been included in previous phylogenomic studies, we sequenced two species of the group, i.e., *C. rodiei* and *S. rubra*. Both species have the Type-III plastome (Fig. 2) which largely agrees with the plastome structure of Neocinnamomeae, Caryodaphnopsideae and the core Lauraceae group [26], with a few exceptions that we will be discussed below. Besides plastome structure, they demonstrated low sequence divergence and high similarity with other species that possess Type-III plastome (≥ 90.1%; Fig. 5; Table S6).

Plastomes of the *Ocotea* group possess considerable variation. All the published *Ocotea* plastomes possess Type-III plastome [29], our newly sequenced samples show different variation and belong to a new type. In *O. bracteosa* and *L. capitata*, the insertion of *trnI-CAU* occurred between *trnL-UAG* and *ccsA* genes in the SSC region (Fig. 2). This variation of gene organization in the SSC has not been reported in plastomes of Lauraceae before. To confirm this unusual variation, we designed specific primers for the inserted *trnI-CAU* and conducted a PCR amplification, confirmed the presence of *trnI-CAU* in the SSC region (Fig. S2). We define this variation as the Type-IV plastome. Notably, the Neotropical dioecious *O. bracteosa* has a plastome structure distinct from two closely related *Ocotea* species (*O. guianensis* and *O. tabacifolia*) belonging to the same dioecious clade in the *Ocotea* group [17, 29, 39], but shows the same plastome type as the monoecious *L. capitata* [40]. This may suggest potential diversity of plastome types in the *Ocotea* group, which is highly probable because the *Ocotea* group is speciose [1]. Moreover, the plastome of *N. angustifolia* was published five years ago [26]. We re-annotated the published plastome of *N. angustifolia*, and found that a *trnI-CAU* gene and a pseudogenizated *rpl23* are inserted in the SSC region; we consider this variation as the Type-V plastome of the family (Fig. 2). The pseudogenizated gene of *rpl23* that has been reported in the genus *Cassytha* [32] was determined because it shows 98% similarity with another *rpl23* gene copy, but differs from the latter in having two internal terminators. More samples representing different lineages of the *Ocotea* group are needed to better understand plastome evolution of this group.

Although we have recognized six plastome types, it is apparent that structural variation may occur within a particular genus or even a certain species. Unusual structural variations of plastomes were found in *Caryodaphnopsis* and *Cam. chartophylla* (≡ *Cin. chartophyllum*; Fig. S5) [28]. The published four plastomes of *Caryodaphnopsis* contain two different types, three of them belong to Type-III (MF939343, MN698962, NC_050345), but one (MF939346) belongs to Type-I as does *Hypodaphnis*. Despite the structural variation, the reported samples of *Caryodaphnopsis* belong to a same clade in the plastome phylogeny [19]. Similar structural variation was found in *Cam. chartophylla*: one sample (OL943972) belongs to Type I while the other one (MW421301) belongs to Type-III [28]. So far, we have found three genera of the family showing infra-generic/specific plastome structural variation, two genera discussed here show reversed plastome variation (Type-I). It remains unclear how and why this exceptional reversal occurs and whether it is rare or common. Without doubt, more samples are needed to verify the structural variation in the future.

### Plastome evolution in Lauraceae

Previous studies have suggested that at least two independent evolutionary events occurred in the plastome evolution of Lauraceae, including different loss events at the IR-LSC boundary [26, 32]. In this study, we found a more complicated evolutionary history and drew a comprehensive picture of plastome evolution of the family Lauraceae by accessing plastome structure of *Hypodaphnis* and the *Mezilaurus* group (Figs. 2 and 7).

The plastome of *Hypodaphnis* is important for an understanding of the plastome evolution of Lauraceae. This genus possesses the Type-I plastome which is similar to that of *Amborella trichopoda* [41] and magnoliids including *Piper* (Piperales), *Liriodendron* and *Magnolia* (Magnoliales), and *Illigera* (Laurales) [26, 30, 42, 43]. The structural similarity of plastomes between *Hypodaphnis* and basal angiosperms suggests that the Type-I plastome structure is ancestral and other types of plastomes of the Lauraceae may have been derived from this type.

The plastomes of Lauraceae show a contracting evolutionary process due to at least two gene loss events at the IR-LSC boundary, followed by an independent expansion of the SSC region in the *Ocotea* group alone (Figs. 2 and 7). The Type-II plastome of Cryptocaryeae may have lost *rpl2* in the IRb region independently due to the contraction of the IRb. For the IR loss of *Cassytha* plastome (Type-VI) after it diverged from the Neocinnamomeae (Type-III), Caryodaphnopsideae (Type-III), the *Mezilaurus* group (Type-III) and the core Lauraceae (Type- III, IV and V), there may have been two scenarios as Wu et al. [32] proposed. One is that contraction of IRa caused the loss of a copy of *rpl2*–*ycf2* in the common ancestor of Type- III, IV, V and VI, and subsequent contractions of the IRa and IRb resulted in the Type-VI plastome with IR completely lost in *Cassytha*. Alternatively, the Type-VI plastome evolved by dropping a copy of the IR region independently, while the common ancestor of Type-III, IV and V lost a copy of *rpl2*–*ycf2* due to contraction of IRa.

Surprisingly, the *Ocotea* group experienced independent expansion events of the SSC region, giving rise to the two newly recognized plastome types, i.e., Type-IV and Type-V (Figs. 2 and 7). Unlike the variation at the IR-LSC boundary in many Lauraceae species, there are three scenarios to explain the type transition from Type-III to Type-IV. First, the insertion of *trnI-CAU* to the SSC region of the ancestral plastome of the *Ocotea* group caused the transition from Type-III to Type-IV. Subsequent insertion of a pseudogenizated *rpl23* gene or a *rpl23* gene to be pseudogenizated may have caused the transition from Type-IV to Type-V in *Nectandra*. Second, the *trnI-CAU_rpl23* segments inserted in the SSC region of the ancestral plastome of the *Ocotea* group, causing the plastome transition from Type-III to Type-V. Subsequent loss of *rpl23* gene resulted in the transition from Type-V to Type-IV. Third, Type-IV and Type-V evolved from Type-III due to the insertion of *trnI-CAU* and *trnI-CAU_rpl23* segments independently. Based on repeats analyses (Fig. 3), we found that the longest repeat (153 bp) occurred in both *O. bracteosa* and *L. capitata*, thereby contributing to the presence of *trnI-CAU* in the SSC region. This result is consistent with the suggestion of Xiao and Ge [28] that longer repeats in the plastomes of the *Ocotea* group than other species of Cinnamomeae may have led to a different evolutionary pattern in this tribe. As the *Ocotea* group is speciose and contains variable plastome types, more plastome patterns and complicated evolutionary histories may be discovered in the future when more species are sampled.

A dated phylogeny is helpful to understand the time frame of the plastome evolution of Lauraceae (Fig. 7). Our age estimates are largely congruent with previous studies [2, 13, 24]. The stem age of the family Lauraceae was in the Early Cretaceous (ca. 107.7 mya). Two independent loss events leading to the transition from Type-III to Type-II and Type-VI in Cryptocaryeae and Cassytheae occurred at ca. 100 mya and ca. 90 mya respectively (Fig. 7), while the expansion event of SSC occurred in the Late Eocene (ca. 38.8 mya; Fig. 7). We have not identified any geological events related to the structural changes of plastomes of the family Lauraceae.

### Phylogenomics of Lauraceae

Our plastid phylogenomic result confirms that the family Lauraceae contains nine major clades corresponding to the eight previously described tribes and the *Mezilaurus* group. The CDS and CPG phylogenies show overall congruent topology except for the tribe Laureae which is one of the most complicated clades with conflicting phylogenetic signals in the plastome evolution [27]. The relationships among the nine clades of Lauraceae are consistent with previous plastome phylogenetic results [19, 20, 29, 44], and receive support from the plastome types as well (Figs. 2 and 7).

*Hypodaphnis* is restricted to tropical Africa and contains only one extant species (i.e., *H. zenkeri*) [2]. Morphologically the genus is the only one with a truly inferior ovary in Lauraceae. According to previous studies based on plastid and nuclear markers, *Hypodaphnis* appears to be sister to all other extant Lauraceae, this position, however, receives rather low support [2, 10, 11]. Song et al. [19] obtained a robust phylogeny of Lauraceae using complete plastomes and nine plastid markers (*matK, psbA-trnH, rbcL, rpl16, rpoB, rpoC1, trnL, trnL‐trnF*, and *trnT‐trnL*) for sampling purposes, and confirmed the sister relationship between *Hypodaphnis* and the remainder of the family with high support. In combination with both morphological and molecular evidence, the clade of *Hypodaphnis* was described as Hypodaphnideae [19]. Here, our new phylogenomic result together with the ancestral plastome type of *Hypodaphnis* corroborate the primitive position of *Hypodaphnis* in the family Lauraceae.

The *Mezilaurus* group is monophyletic and consists of six genera including *Anaueria*, *Chlorocardium*, *Clinostemon*, *Mezilaurus*, *Sextonia* and *Williamodendron* [2]. This group is sister to the core Lauraceae clade according to previous molecular studies based on nuclear and plastid markers [2, 10, 11, 19, 45]. Our phylogenomic tree confirms that the *Mezilaurus* group is monophyletic and the sister relationship of this group to the core Lauraceae clade receives high support (Fig. 6, S4). However, no synapomorphy has been recorded in morphology and anatomy of the clade to date due to high variability [45, 46]. Neither does the plastome structure provide useful taxonomic characters to unite all genera of this group together. Further studies are necessary to better understand the synapomorphy of the group.

### Herbariomics in Lauraceae

Phylogenetic studies of Lauraceae are still in their early stages due to the lack of plant materials. Global herbaria house numerous accurately identified plant specimens and are a potential material source for species sampling [34, 35]. Herbariomics and genome skimming based on NGS technique offer a powerful, efficient, and promising approach to obtain more species and DNA sequences [33, 34]. Museum specimens usually contains low DNA quality because of degradation and fragmentation and tissues of Lauraceae are rich in polysaccharides and polyphenols [1, 34]. These factors limit full use of herbarium specimens in phylogenetic studies of Lauraceae [1, 35]. In this study, we suggest that the mCTAB method is sufficient for extracting DNA from herbarium samples of Lauraceae, 20–30 mg leaf tissues of herbarium specimens can produce over 1,000 ng DNA (Table 2) [47]. We successfully obtained five plastomes of Lauraceae using specimens collected 15 years ago (Table 2), and filled the sampling gap for the phylogeny of the Lauraceae by adding plastomes of Hypodaphnideae and the *Mezilaurus* group. Our study suggests that herbariomics provides a new opportunity and opens a new era for plastome phylogenomic studies of Lauraceae.

**Table 2:** Vouchers and accession nos. of five new sequenced plastomes in this study

Latin Name	*Licaria* *capitata*	*Ocotea* *bracteosa*	*Chlorocardium* *rodiei*	*Sextonia* *rubra*	*Hypodaphnis zenkeri*
Collection	R. Acevedo & R. Acosta 878	E.L. Taylor & al. E1123	K.M. Redden 5196	C. Brewer-Carias s.n.	J.D. Idennedy 1553
Locality	Mexico: Veracruz	Brazil: Maranhao	Guyana	Venezuela	Nigeria
Collection time	1986	1983	2007	1991	-
Herbarium	A	GH	MO	MO	A
DNA yield (ng)	1611.4	517.2	1087.8	1222.7	1805.1
Barcode	-	-	MO-2,195,744	MO-252,354	-
Identification	R. Acevedo R.	J. Rowher	s.n.	H. van der Werff	s.n.

## Conclusion

Utilizing leaf tissue of herbarium specimens, we successfully obtained five new plastomes of Lauraceae, representing five genera (*Licaria*, *Ocotea, Chlorocardium*, *Sextonia* and *Hypodaphnis*) belonging to three different clades of the family, i.e., Hypodaphnideae, the *Mezilaurus* group, and the *Ocotea* group. *Hypodaphnis* possesses the ancestral plastome type of the family with *rpl2*, *rpl23* and *trnI-CAU* duplicated in the IR region. The *Mezilaurus* group possesses the same plastome type as the core Lauraceae group. Two new plastome types of the family Lauraceae were recognized in the *Ocotea* group. *Licaria capitata* and *O. bracteosa* possess plastomes with *trnI-CAU* inserted between *trnL-UAG* and *ccsA* in the SSC region (Type-IV) unlike their relatives, whereas *N. angustifolia* has a plastome with *trnI-CAU* and pseudogenizated *rpl23* inserted in the same region (Type-V). Plastome evolution of Lauraceae has become better understood by adding plastomes of *Hypodaphnis* and the *Mezilaurus* group in phylogenomic studies and filling the sampling gap of unusual lineages of the family Lauraceae. We also show that herbariomics is a powerful tool to obtain extensive species sampling from accurately identified herbarium specimens for phylogenetic studies of such a difficult family as the Lauraceae.

## Materials and methods

### Taxon sampling

We obtained leaf samples of *L. capitata*, *O. bracteosa*, *C. rodiei*, *S. rubra* and *H. zenkeri* from herbarium specimens deposited in the Herbarium of Missouri Botanical Garden (MO) and Harvard University Herbaria (A, GH) (Table 2). To infer the plastome phylogeny of Lauraceae, plastome sequences of the family were also downloaded from NCBI (accessed October 13 2021). In general, we downloaded one plastome sequence for each genus of the family when available. Multiple sequences of genera in Laureae with ambiguous phylogenetic relationships were selected according to Song et al. [26]. In total 35 plastomes were selected, included 31 genera representing all nine clades of Lauraceae. *Calycanthus chinensis*, *Chimonanthus nitens* and *Chim. praecox* (Calycanthaceae, Laurales) were chosen as the outgroup. Information of sequences and their accession numbers are listed in Table S7.

### DNA extraction and genomic sequencing

Genomic DNA was extracted from 20 to 30 mg leaves of herbarium specimens using a modified CTAB method (mCTAB) [47]. 3% CTAB was used, and approximately 2% polyvinyl polypyrrolidone (PVP) and 0.1% β-mereaptoethanol were added. In order to make full use of leaf materials, DNA extraction was repeated once, and DNA solutions were combined at the end. DNA quality was assessed with Agilent 5400 (Agilent Technologies Inc., U.S.A.). Short-insert libraries were prepared following the manufacturer’s manual (Illumina) without a supersonic fragmentation treatment of the total DNA considering the degraded nature of herbarium specimens with short fragments. The DNA libraries were sequenced by Illumina Novo Seq6000 at Novogene Co., Ltd (Beijing, China). A total of ~ 2 Gb of 150 bp paired-end reads were obtained for each sample.

### Genome assembly and annotation

GetOrganelle 1.7.5.0 [48] was used for plastome assembly. GetOrganelle integrates SPAdes 3.13.0 [49], Bowtie2 2.4.4 [50], BLAST + 2.5.0 [51] were applied to assemble plastomes *de novo*. Plastomes were annotated using GeSeq [52] followed by manual adjustment in Geneious Prime 2020.0.5. Sequences downloaded from online database were annotated again to avoid potential annotation errors, and ambiguous genes were double-checked by CpGAVAS2 [53]. *Cinnamomum japonicum* (MT621639) and *Beilschmiedia appendiculata* (NC_051896) were selected as references for species of the core Lauraceae group and other sampled species of Lauraceae separately. All plastomes were adjusted to start at *trnH-GUG* gene for downstream phylogenetic analyses and plastome structure comparison. Circular genome maps were drawn by OrganellarGenomeDRAW tool 1.3.1 (OGDRAW) [54] and CpGAVAS2, then edited in Photoshop 2020.

### Genome structure identification

To verify the structure of the newly sequenced plastomes, a pair of gene-specific primers (1-F: GCCGCCATGGTGAAATTGGTAGA, 1-R: GCATCCATRGCTGAATGGTTAAAG) were designed to determine the presence of *trnI-CAU* in *L. capitata* and *O. bracteosa*. *Sextonia rubra* was selected as a control (Fig. S2A). PCR was performed in 50 µL reaction mixtures containing 25 µL of 2× Mix Buffer, 1 µL of 10 μm of each primer, 22 µL of ddH_2_O and 1 µL template DNA, and programmed in Applied Biosystems 9700 Thermal cycler (Thermo Fisher Scientific, MA, USA) with an initial denaturation at 95 °C for 5 min, then 35 cycles at 95 °C for 30 s, 55 °C for 30 s and 72 °C for 60 s, followed by a final extension of 72 °C for 2 min. The 2 µL PCR product was separated using 1% agarose gel stained with Super GelBlue™ (UElandy, Suzhou, China) staining solution in 1X tris acetate ethylenediaminetetraacetic acid. The image of the gel was digitized using Tanon 2500 (Tanon, Shanghai, China). All steps above were conducted at Springen Biotechnology (Nanjing, China).

### Phylogenetic analyses

Phylogenetic analyses were conducted using both a complete plastome (CPG) dataset and a 79 protein-coding genes (CDS) dataset. The CPG dataset was aligned with MAFFT 7.480 [55] using “-auto” strategy, with ambiguously aligned fragments removed using Gblocks 0.91b [56]. CDS gene extraction was performed using the script ‘get_annotated_regions_from_gb.py’ of Jin [57], then the CDS dataset was aligned using MAFFT with “L-INS-i” strategy. The multiple sequence alignment was visualized using BioEdit 7.2.5 [58]. Gap sites of CDS genes were removed with trimAl 1.4.1 [59] using “-automated1” strategy, then only genes more than 100 bp long were concatenated into a matrix by PhyloSuite v1.2.2 [60]. Phylogenetic trees were inferred based on CPG and CDS datasets using Maximum likelihood (ML) method in IQ-TREE 2.1.2 [61] under Edge-linked partition model and TVM + F + I + G4 model determined by ModelFinder [62] according to the best Bayesian Information Criterion (BIC) score, respectively. Support value accessed with 5,000 ultrafast bootstraps (UFboot) replicates [63] and 1,000 SH-like approximate likelihood ratio test (SH-aLRT) [64] replicates. Clades were considered as reliable when their SH-aLRT ≧ 80% and UFboot ≧ 95%.

### Repeat sequence analyses

Repeat sequence analyses of the five newly sequenced plastomes were generated by CpGAVAS2. Vmatch 2.2.1 [65] was used to detect long repeats (-f -p -l 30 -identity 90 -h 3). Long Tandem Repeats (size of repeat unit ≧ 7) identified with the online Tandem Repeats Finder 3.01 (TRF) [66], parameters were set as 2 7 7 80 10 50 500 -f -d -m. MIcroSAtellite identification tool v2.1 (MISA) [67] was implemented to identify simple sequence repeats (SSRs) in the chloroplast genomes (1–10 2–6 3–5 4–5 5–5 6 − 5).

### Genome structure analysis and genome comparisons

Plastomes of Lauraceae can be better understood with structural analyses and comparisons of genomes. We first calculated pairwise distance among genera based on the complete sequences and visualized the similarity via a hot map generated on ImageGP website [68]. Then we calculated the percentage of variable sites among coding and non-coding regions to visualize the variations at gene level. The violin plot was generated by R package ggplot2 3.3.5 [69]. Five newly sequenced plastomes and eight species covering the nine clades of Lauraceae were selected for structural comparison. Because plastome structure was already explored in Perseeae and Laureae [19, 32], only *Persea americana* was chosen as representative here. More than one sequence was selected from Cinnamomeae to compare plastome structure among them.

### Divergence time estimation

The ML tree generated by the concatenated CDS dataset was used for dating analyses. We selected five macrofossils for calibration following Li et al. [24]. First, the middle Albian fossil *Virginianthus calycanthoides* was employed to calibrate the crown age of Laurales at the root node of the tree. We defined a minimum age of 107.7 mya according to Massoni et al. [70] and set the upper boundary age 113 mya of Albian as the maximum age of this node (C1: age 107.7–113 mya). Second, *Jerseyanthus calycanthoides* was used to calibrate the split between *Calycanthus* and *Chimonanthus*. The age of this fossil was believed to be from the Coniacian-Santonian boundary (C2: age 85.8–86.8 mya) [70]. Third, the Cretaceous fossil taxon *Neusenia tetrasporangiata* was applied to calibrate the stem age of *Neocinnamomum*, the boundary age of Santonian-Campanian (C3: age 72.1–86.3 mya) [71] was set as the age range of the fossil. Fourth, *Alseodaphne changchangensis* was applied to calibrate the crown age of the *Persea* group. The age of this fossil was dated back to the late Early Eocene to the early Late Eocene (C4: age 37–48 mya) [72]. Fifth, *Machilus maomingensis* was used to calibrate the stem age of *Machilus*. The locality of this fossil was dated to the Eocene-Oligocene boundary (C5: age 33.7–33.9 mya) [73].

Dating analyses were carried out with the approximate likelihood calculation using MCMCTree in PAML4.9j [74]. The time unit was set to 100 mya, and the default soft tail of 2.5% was applied for the minimum and maximum bounds of all calibration points. For the root node and nodes whose age were well estimated (C1, C2 and C5), we used the lower and upper bounds that can be set to place the maximum probability of the node falling in a certain space between the calibrations. The remaining calibration nodes (C3 and C4) were used for the lower minimal bound with offset (p) and scale parameter (c) set as 0.1 and 0.2, respectively. The substitution rate was a rough estimation using BASEML (in PAML) at first. Then the ML estimates of branch lengths, the gradient vector, and Hessian matrix were calculated in MCMCTree using the GTR + G substitution models (model = 7). The parameter of rgene_gamma and sigma2_gamma was set as G (1, 33.3) and G (1, 4.5) according to previous estimation, respectively. A relaxed-clock model (clock = 2) was established. Two independent MCMC runs were conducted with burnin = 2,000,000, sampfreq = 100, nsample = 100,000. The stationary state and convergence of each run were checked in Tracer v.1.7.1 [75] to ensure that all parameters had effective sample sizes (ESS) above 200.

### Electronic supplementary material

Below is the link to the electronic supplementary material.

**Supplementary Material 1: Fig. S1**. Circular gene map of the five newly sequenced plastomes.

**Supplementary Material 2: Fig. S2**. Validation of the presence of *trnI-CAU* in the plastomes of *Licaria capitata* and *Ocotea bracteosa*.

**Supplementary Material 3: Fig. S3**. Percentages of variable characters in 35 aligned Lauraceae plastomes

**Supplementary Material 4: Fig. S4**. Maximum-likelihood (ML) tree inferred from the complete plastomes.

**Supplementary Material 5: Fig. S5**. Unusual plastomes in *Caryodaphnopsis* and* Camphora*.

**Supplementary Material 6: Table S1**. Summary of available plastomes of Lauraceae in NCBI. **Table S2**. Number of three repeats types. **Table S3**. Length of three repeats types. **Table S4**. Types and amounts of SSRs in the five newly sequenced chloroplast genomes. **Table S5**. The distribution of simple sequence repeats. **Table S6**. The pairwise similarity of Lauraceae. **Table S7**. Plastomes and sequences obtained from NCBI for phylogenetic studies.

## Data Availability

All five newly sueqenced and annotated plastomes generated and analysed in this study are available in the NCBI repository with accession number from OQ621667 to OQ621671. Accession numbers of all plastomes used in this study can be found in Additional file 6: Table S7.

## References

[1] Rohwer JG, Kubitzki K, Rohwer JG, Bittrich V (1993). Lauraceae. The families and genera of vascular plants.

[2] Chanderbali AS, van der Werff H, Renner SS (2001). Phylogeny and historical biogeography of Lauraceae: evidence from the chloroplast and nuclear genomes. Ann Mo Bot Gard.

[3] Angiosperm Phylogeny Group (2016). An update of the Angiosperm Phylogeny Group classification for the orders and families of flowering plants: APG IV. Bot J Linn Soc.

[4] van der Werff H, Richter HG (1996). Toward an improved classification of Lauraceae. Ann Mo Bot Gard.

[5] Gentry AH (1988). Changes in plant community diversity and floristic composition on environmental and geographical gradients. Ann Mo Bot Gard.

[6] Tang CQ, Tang CQ (2015). Evergreen broad-leaved forests. The subtropical vegetation of Southwestern China: plant distribution, Diversity and Ecology.

[7] Zheng WY, Zeng WH, Tang YS, Shi W, Cao KF (2019). Species diversity and biogeographical patterns of Lauraceae and Fagaceae in northern tropical and subtropical regions of China. Acta Ecol Sin.

[8] Kostermans AJGH (1957). Lauraceae Reinwardtia.

[9] Li HW, Li J, Huang PH, Wei FN, Cui HB, van der Werff H, Wu ZY, Raven PH, Hong DY (2008). Lauraceae. Flora of China.

[10] Rohwer JG (2000). Toward a phylogenetic classification of the Lauraceae: evidence from *matK* sequences. Syst Bot.

[11] Rohwer JG, Rudolph B (2005). Jumping genera: the phylogenetic positions of *Cassytha*, *Hypodaphnis*, and *Neocinnamomum* (Lauraceae) based on different analyses of *trnK* intron sequences. Ann Mo Bot Gard.

[12] Li J, Conran JG, Christophel DC, Christophel DC, Li ZM, Li L (2008). Phylogenetic relationships of the *Litsea* complex and core Laureae (Lauraceae) using ITS and ETS sequences and morphology. Ann Mo Bot Gard.

[13] Li L, Li J, Rohwer JG, van der Werff H, Wang ZH, Li HW (2011). Molecular phylogenetic analysis of the *Persea* group (Lauraceae) and its biogeographic implications on the evolution of tropical and subtropical Amphi-Pacific disjunctions. Am J Bot.

[14] Rohwer JG, Li J, Rudolph B, Schmidt SA, van der Werff H, Li HW (2009). Is *Persea* (Lauraceae) monophyletic? Evidence from nuclear ribosomal ITS sequences. Taxon.

[15] Huang JF, Li L, van der Werff H, Li HW, Rohwer JG, Crayn DM (2016). Origins and evolution of cinnamon and camphor: a phylogenetic and historical biogeographical analysis of the *Cinnamomum* group (Lauraceae). Mol Phylogenet Evol.

[16] Mo YQ, Li L, Li JW, Rohwer JG, Li HW, Li J (2017). *Alseodaphnopsis*: a new genus of Lauraceae based on molecular and morphological evidence. PLoS ONE.

[17] Trofimov D, de Moraes PLR, Rohwer JG. Towards a phylogenetic classification of the *Ocotea* complex (Lauraceae): classification principles and reinstatement of *Mespilodaphne*, Bot J Linn Soc 2019:19025–50.

[18] Trofimov D, Rohwer JG (2020). Towards a phylogenetic classification of the *Ocotea* complex (Lauraceae): an analysis with emphasis on the Old World taxa and description of the new genus *Kuloa*. Bot J Linn Soc.

[19] Song Y, Yu WB, Tan YH, Jin JJ, Wang B, Yang JB (2020). Plastid phylogenomics improve phylogenetic resolution in the Lauraceae. J Syst Evol.

[20] Liu ZF, Ma H, Ci XQ, Li L, Song Y, Liu B (2021). Can plastid genome sequencing be used for species identification in Lauraceae? Bot. J Linn Soc.

[21] Tian Y, Zhou J, Zhang Y, Wang S, Wang Y, Liu H (2021). Res progress plant Mol Syst Lauraceae Biology (Basel).

[22] Ruhlman TA, Jansen RK, Maliga P (2014). The plastid genomes of flowering plants. Chloroplast Biotechnology.

[23] Gitzendanner MA, Soltis PS, Yi TS, Li DZ, Soltis DE (2018). Plastome phylogenetics: 30 years of inferences into plant evolution. Adv Bot Res.

[24] Li HW, Liu B, Davis CC, Yang Y (2020). Plastome phylogenomics, systematics, and divergence time estimation of the *Beilschmiedia* group (Lauraceae). Mol Phylogenet Evol.

[25] Li HT, Luo Y, Gan L, Ma PF, Gao LM, Yang JB (2021). Plastid phylogenomic insights into relationships of all flowering plant families. BMC Biol.

[26] Song Y, Yu WB, Tan YH, Liu B, Yao X, Jin JJ (2017). Evolutionary comparisons of the chloroplast genome in Lauraceae and insights into loss events in the Magnoliids. Genome Biol Evol.

[27] Xiao TW, Xu Y, Jin L, Liu TJ, Yan HF, Ge XJ (2020). Conflicting phylogenetic signals in plastomes of the tribe Laureae (Lauraceae). PeerJ.

[28] Xiao TW, Ge XJ (2022). Plastome structure, phylogenomics, and divergence times of tribe Cinnamomeae (Lauraceae). BMC Genomics.

[29] Trofimov D, Cadar D, Schmidt-Chanasit J, Rodrigues de Moraes PL, Rohwer JG (2022). A comparative analysis of complete chloroplast genomes of seven *Ocotea* species (Lauraceae) confirms low sequence divergence within the *Ocotea* complex. Sci Rep.

[30] Zhu A, Guo W, Gupta S, Fan W, Mower JP (2016). Evolutionary dynamics of the plastid inverted repeat: the effects of expansion, contraction, and loss on substitution rates. New Phytol.

[31] Simmonds SE, Smith JF, Davidson C, Buerki S (2021). Phylogenetics and comparative plastome genomics of two of the largest genera of angiosperms, *Piper* and *Peperomia* (Piperaceae). Mol Phylogenet Evol.

[32] Wu CS, Wang TJ, Wu CW, Wang YN, Chaw SM (2017). Plastome evolution in the sole hemiparasitic genus laurel dodder (*Cassytha*) and insights into the plastid phylogenomics of Lauraceae. Genome Biol Evol.

[33] Staats M, Erkens RH, van de Vossenberg B, Wieringa JJ, Kraaijeveld K, Stielow B et al. Genomic treasure troves: complete genome sequencing of herbarium and insect museum specimens. PLoS ONE. 2013:8:e69189. 10.1371/journal.pone.0069189 PMC3726723 23922691

[34] Kates HR, Doby JR, Siniscalchi CM, LaFrance R, Soltis DE, Soltis PS (2021). The effects of herbarium specimen characteristics on short-read NGS sequencing success in nearly 8000 specimens: old, degraded samples have lower DNA yields but consistent sequencing success. Front Plant Sci.

[35] Bakker FT, Lei D, Yu J, Mohammadin S, Wei Z, van de Kerke S (2015). Herbarium genomics: plastome sequence assembly from a range of herbarium specimens using an iterative organelle genome assembly pipeline. Bot J Linn Soc.

[36] Dodsworth S, Guignard MS, Christenhusz MJ, Cowan R, Knapp S, Maurin O (2008). Potential of herbariomics for studying repetitive DNA in angiosperms. Front Ecol Evol.

[37] Nevill PG, Zhong X, Tonti-Filippini J, Byrne M, Hislop M, Thiele K (2020). Large scale genome skimming from herbarium material for accurate plant identification and phylogenomics. Plant Methods.

[38] Zeng CX, Hollingsworth PM, Yang J, He ZS, Zhang ZR, Li DZ (2018). Genome skimming herbarium specimens for DNA barcoding and phylogenomics. Plant Methods.

[39] Meissner CF. Lauraceae. In: de Candolle AP, editor Prodromus Systematis Regni Vegetabilis, vol. 15. Paris: Sumptibus Sociorum Treuttel et Würtz; 1864. p. 1–522.

[40] Kostermans AJGH (1937). Revision of the Lauraceae II. The genera *Endlicheria*, *Cryptocarya* (american species) and *Licaria*. Meded Bot Mus Herb Rijks Univ Utrecht.

[41] Goremykin VV, Hirsch-Ernst KI, Wolfl S, Hellwig FH (2003). Analysis of the *Amborella trichopoda* chloroplast genome sequence suggests that *Amborella* is not a basal angiosperm. Mol Biol Evol.

[42] Cai Z, Penaflor C, Kuehl JV, Leebens-Mack J, Carlson JE, dePamphilis CW (2016). Complete plastid genome sequences of *Drimys*, *Liriodendron*, and *Piper*: implications for the phylogenetic relationships of magnoliids. BMC Evol Biol.

[43] Xin YX, Xin J, Yao GQ, Qu YY, Feng FY, Song Y (2020). The complete chloroplast genome sequence of *Illigera celebica*. Mitochondrial DNA B Resour.

[44] Yang Z, Liu B, Yang Y, Ferguson DK (2022). Phylogeny and taxonomy of *Cinnamomum* (Lauraceae). Ecol. Evol.

[45] Alves FM, Souza VC (2013). Phylogenetic analysis of the neotropical genus *Mezilaurus* and reestablishment of *Clinostemon* (Lauraceae). Taxon.

[46] Vaz PP, Alves FM, Arruda RDD (2019). Systematic implications of leaf anatomy in the neotropical *Mezilaurus* clade (Lauraceae). Bot J Linn Soc.

[47] Li JL, Wang S, Yu J, Wang L, Zhou SL (2013). A modified CTAB protocol for plant dna extraction. Chin Bull Bot.

[48] Jin JJ, Yu WB, Yang JB, Song Y, dePamphilis CW, Yi TS (2020). GetOrganelle: a fast and versatile toolkit for accurate de novo assembly of organelle genomes. Genome Biol.

[49] Bankevich A, Nurk S, Antipov D, Gurevich AA, Dvorkin M, Kulikov AS (2012). SPAdes: a new genome assembly algorithm and its applications to single-cell sequencing. J Comput Biol.

[50] Langmead B, Salzberg SL (2012). Fast gapped-read alignment with Bowtie 2. Nat. Methods.

[51] Camacho C, Coulouris G, Avagyan V, Ma N, Papadopoulos J, Bealer K (2009). BLAST+: architecture and applications. BMC Bioinformatics.

[52] Tillich M, Lehwark P, Pellizzer T, Ulbricht-Jones ES, Fischer A, Bock R (2017). GeSeq- versatile and accurate annotation of organelle genomes. Nucleic Acids Res.

[53] Shi L, Chen H, Jiang M, Wang L, Wu X, Huang L (2019). CPGAVAS2, an integrated plastome sequence annotator and analyzer. Nucleic Acids Res.

[54] Lohse M, Drechsel O, Kahlau S, Bock R (2013). OrganellarGenomeDRAW-a suite of tools for generating physical maps of plastid and mitochondrial genomes and visualizing expression data sets. Nucleic Acids Res.

[55] Katoh K, Standley DM (2013). MAFFT multiple sequence alignment software version 7: improvements in performance and usability. Mol Biol Evol.

[56] Talavera G, Castresana J (2007). Improvement of phylogenies after removing divergent and ambiguously aligned blocks from protein sequence alignments. Syst Biol.

[57] Jin JJ, PersonalUtilities. 2019. https://github.com/Kinggerm/PersonalUtilities. Accessed 8 January 2020.

[58] Hall TA, BioEdit. A user-friendly biological sequence alignment editor and analysis program for Windows 95/98/NT. Nucleic Acids Symp. Ser. 1999;41:95 – 98.

[59] Capella-Gutierrez S, Silla-Martinez JM, Gabaldon T (2009). trimAl: a tool for automated alignment trimming in large-scale phylogenetic analyses. Bioinformatics.

[60] Zhang D, Gao FI, Jakovlić I, Zou H, Zhang J, Li WX (2020). PhyloSuite: an integrated and scalable desktop platform for streamlined molecular sequence data management and evolutionary phylogenetics studies. Mol Ecol Resour.

[61] Nguyen LT, Schmidt HA, von Haeseler A, Minh BQ (2015). IQ-TREE: a fast and effective stochastic algorithm for estimating maximum-likelihood phylogenies. Mol Biol Evol.

[62] Kalyaanamoorthy S, Minh BQ, Wong TKF, von Haeseler A, Jermiin LS (2017). ModelFinder: fast model selection for accurate phylogenetic estimates. Nat Methods.

[63] Minh BQ, Nguyen MA, von Haeseler A (2013). Ultrafast approximation for phylogenetic bootstrap. Mol Biol Evol.

[64] Guindon S, Dufayard JF, Lefort V, Anisimova M, Hordijk W, Gascuel O (2010). New algorithms and methods to estimate maximum-likelihood phylogenies: assessing the performance of PhyML 3.0. Syst. Biol.

[65] Kurtz S. The Vmatch large scale sequence analysis software. 2017. http://www.vmatch.de/. Accessed 11 November 2021.

[66] Benson G (1999). Tandem repeats finder: a program to analyze DNA sequences. Nucleic Acids Res.

[67] Beier S, Thiel T, Münch T, Scholz U, Mascher M (2017). MISA-web: a web server for microsatellite prediction. Bioinformatics.

[68] Chen T, Liu YX, Huang L (2022). ImageGP: an easy-to-use data visualization web server for scientific researchers. iMeta.

[69] Wickham H (2016). ggplot2: elegant graphics for data analysis.

[70] Massoni J, Doyle J, Sauquet H (2015). Fossil calibration of Magnoliidae, an ancient lineage of angiosperms. Palaeontol Electron.

[71] Eklund H (2000). Lauraceae flowers from the late cretaceous of North Carolina, U.S.A. Bot J Linn Soc.

[72] Li JZ, Qiu J, Liao WB, Jin JH (2009). Eocene fossil *alseodaphne* from Hainan Island of China and its paleoclimatic implications. Sci China Ser D-Earth Sci.

[73] Li L, Madriñán S, Li J (2016). Phylogeny and biogeography of *Caryodaphnopsis* (Lauraceae) inferred from low-copy nuclear gene and ITS sequences. Taxon.

[74] Yang ZH (2007). PAML 4: phylogenetic analysis by maximum likelihood. Mol Biol Evol.

[75] Rambaut A, Drummond AJ, Xie D, Baele G, Suchard MA (2018). Posterior summarization in bayesian phylogenetics using Tracer 1.7. Syst. Biol.

