# Supplementary material for: New insights into the plastome evolution of Lauraceae using herbariomics
Source: BMC Plant Biol. 2023 Aug 10;23:387. doi: 10.1186/s12870-023-04396-4 (PMC10413609; doi:10.1186/s12870-023-04396-4)
Supplement: Supplementary file 1 — Supplementary Material 1: Fig. S1. Circular gene map of the five newly sequenced plastomes. [file 12870_2023_4396_MOESM1_ESM.pdf]

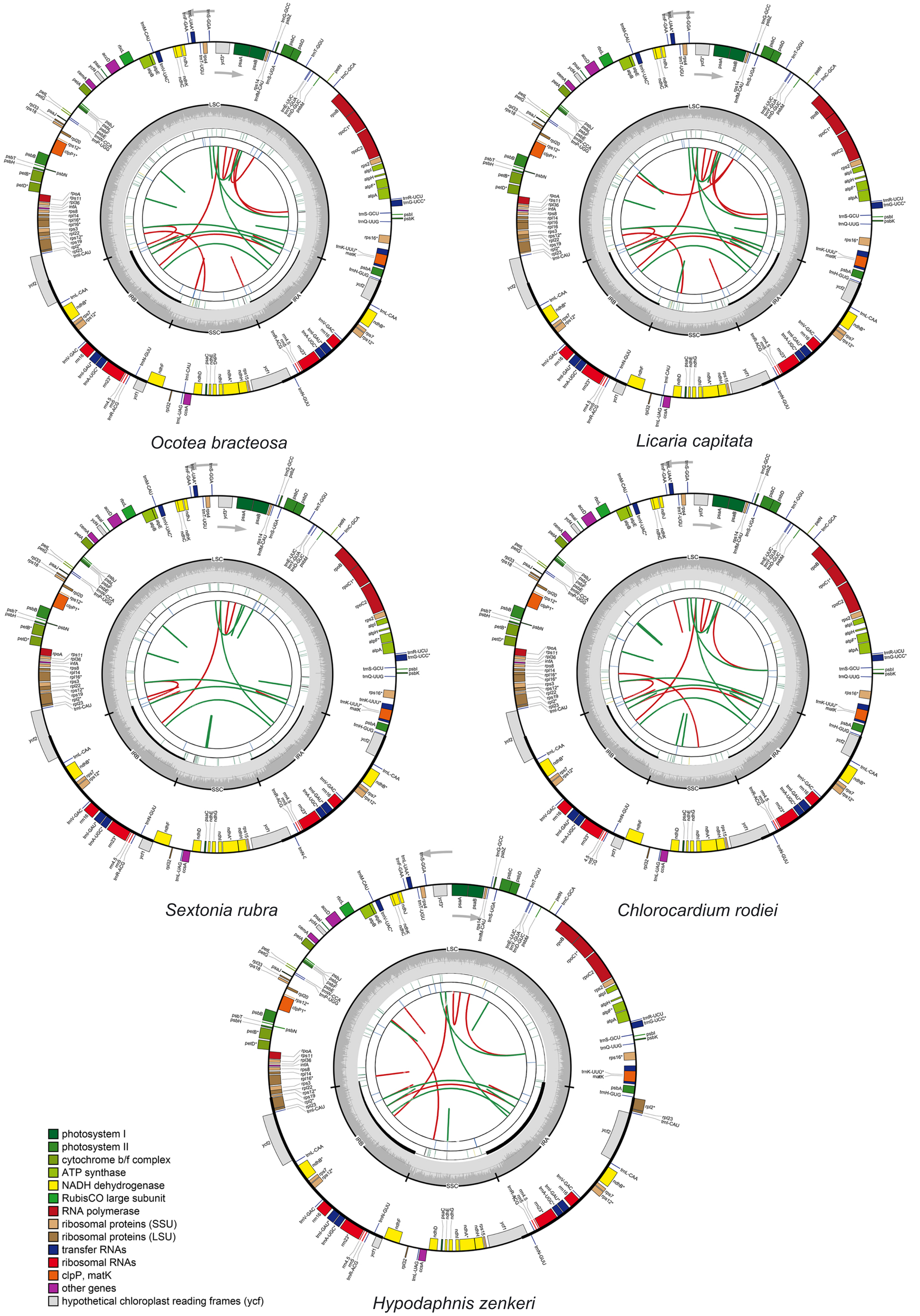

Fig. S1. Circular gene map of the five newly sequenced plastomes. The map contains five rings. From the center going outward, the first circle shows the forward and reverse repeats connected with red and green arcs respectively. The next circle illustrates the tandem repeats marked with short bars. The third circle shows the microsatellite sequences identified using MISA. The fourth circle indicates small single copy (SSC), large single copy (LSC), and inverted repeats (IRa, IRb). The dashed dark gray area indicates the GC content of the plastome, while the light gray area shows the AT content. The last circle displays the gene structure on the plastome. Genes drawn outside the circle are transcribed in a counterclockwise direction, those inside are transcribed in a clockwise direction. The genes were colored based on their functional categories.
