# Supplementary material for: New insights into the plastome evolution of Lauraceae using herbariomics
Source: BMC Plant Biol. 2023 Aug 10;23:387. doi: 10.1186/s12870-023-04396-4 (PMC10413609; doi:10.1186/s12870-023-04396-4)

Fig. S2. Validation of the presence of *trnI-CAU* in the plastomes of *Licaria capitata* and *Ocotea bracteosa*. A. Position of the gene-specific primers in the plastomes of *Licaria capitata* and *Ocotea bracteosa*. B. Polymerase chain reaction result visualization using agarose gel electrophoresis. L, *Licaria capitate*; O, *Ocotea bracteosa*; S, *Sextonia rubra*; M, maker (100-2,000 bp).

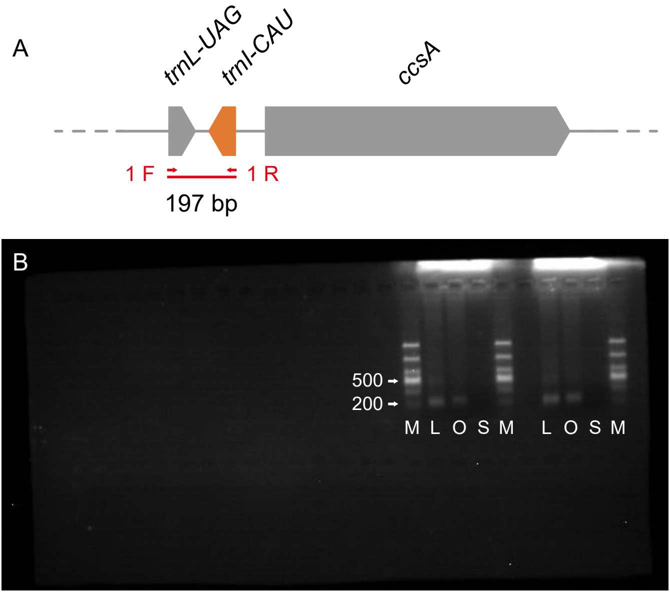

Supplement: Supplementary file 2 — Supplementary Material 2: Fig. S2. Validation of the presence of trnI-CAU in the plastomes of Licaria capitata and Ocotea bracteosa. [file 12870_2023_4396_MOESM2_ESM.pdf]
