# Supplementary material for: New insights into the plastome evolution of Lauraceae using herbariomics
Source: BMC Plant Biol. 2023 Aug 10;23:387. doi: 10.1186/s12870-023-04396-4 (PMC10413609; doi:10.1186/s12870-023-04396-4)
Supplement: Supplementary file 3 — Supplementary Material 3: Fig. S3. Percentages of variable characters in 35 aligned Lauraceae plastomes [file 12870_2023_4396_MOESM3_ESM.pdf]

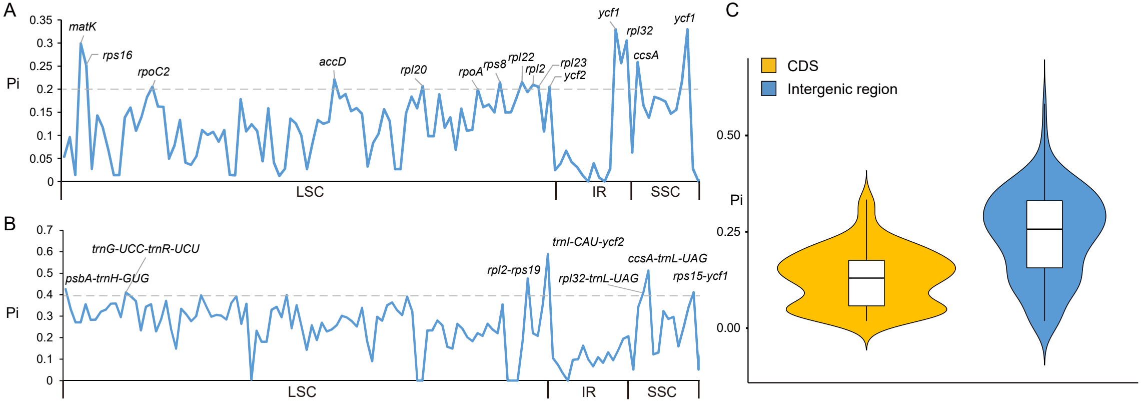

Fig. S3. Percentages of variable characters in 35 aligned Lauraceae plastomes. A. Coding regions, the loci with pi over 0.2 are indicated; B. Noncoding regions, the loci with pi over 0.4 are indicated; C. Analysis of the differences between coding and intergenic region. Y-axis: nucleotide diversity of each locus.
