# Supplementary material for: New insights into the plastome evolution of Lauraceae using herbariomics
Source: BMC Plant Biol. 2023 Aug 10;23:387. doi: 10.1186/s12870-023-04396-4 (PMC10413609; doi:10.1186/s12870-023-04396-4)
Supplement: Supplementary file 4 — Supplementary Material 4: Fig. S4. Maximum-likelihood (ML) tree inferred from the complete plastomes. [file 12870_2023_4396_MOESM4_ESM.pdf]

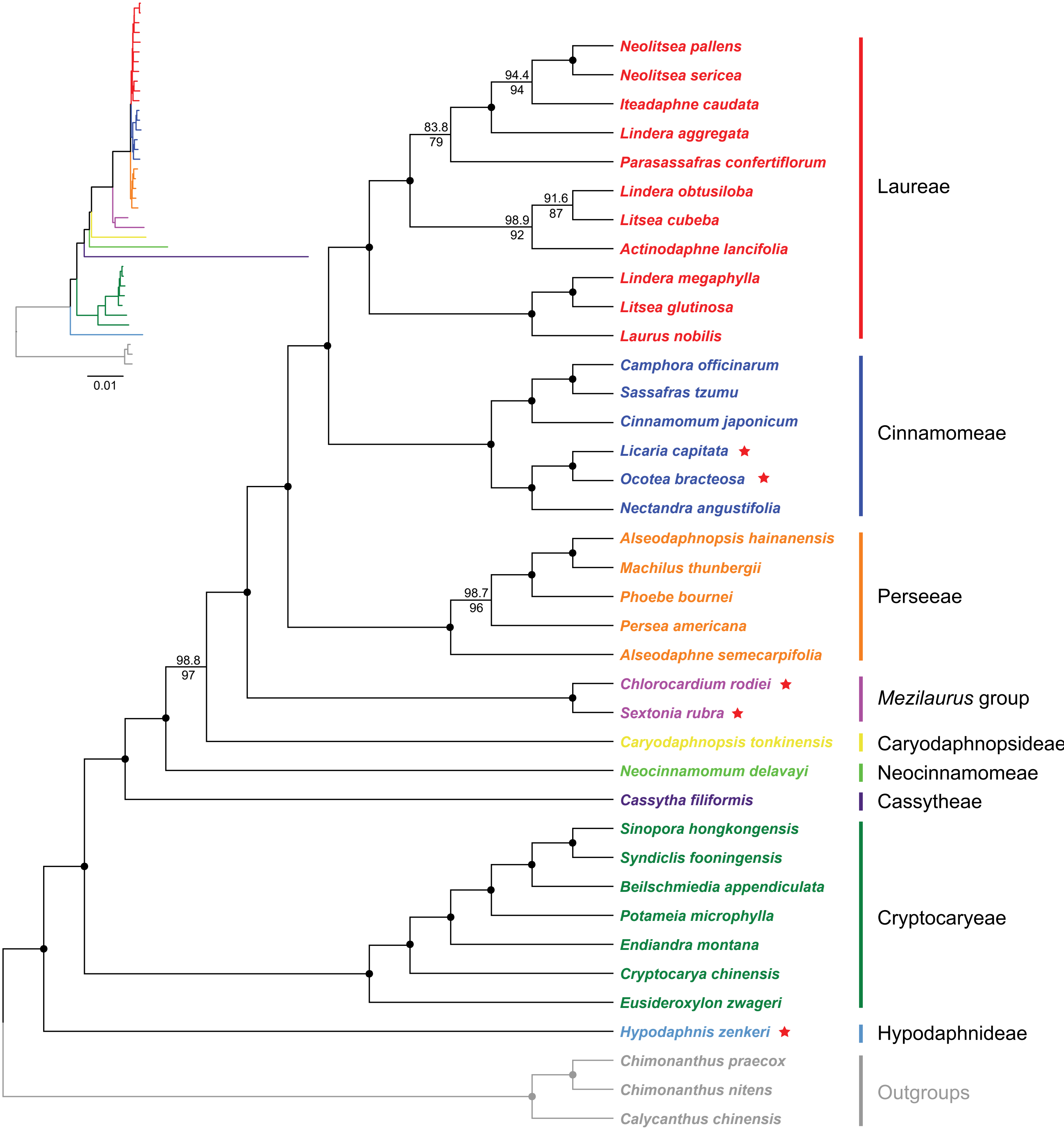

Fig. S4. Maximum-likelihood (ML) tree inferred from the complete plastomes. Different tribes are highlighted with different colors. The five newly sequenced species are indicated with a red star. Each branch is assigned with UFBoot and SH -aLRT supports that are drawn above and below the line, respectively. The clade with 100% supports for both tests are displayed by a black circle at node. The phylogenetic tree with branch length is showed on the upper left.
