# Supplementary figures and images for: New insights into the plastome evolution of Lauraceae using herbariomics
Source: BMC Plant Biol. 2023 Aug 10;23:387. doi: 10.1186/s12870-023-04396-4 (PMC10413609; doi:10.1186/s12870-023-04396-4)

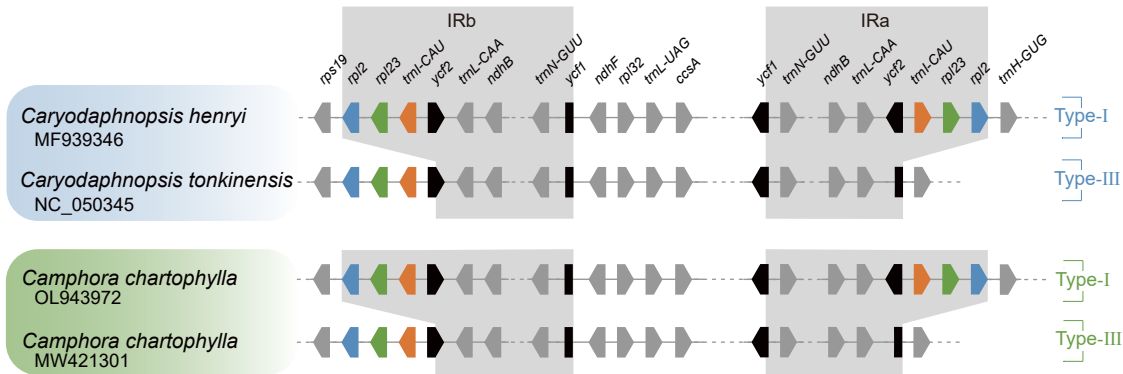

**Fig. S5.** Unique plastomes in *Caryodaphnopsis* and *Camphora*.

Supplement: Supplementary file 5 — Supplementary Material 5: Fig. S5. Unusual plastomes in Caryodaphnopsis and Camphora. [file 12870_2023_4396_MOESM5_ESM.pdf]
